# The Palaeoenvironmental Impact of Prehistoric Settlement and Proto-Historic Urbanism: Tracing the Emergence of the Oppidum of Corent, Auvergne, France

**DOI:** 10.1371/journal.pone.0121517

**Published:** 2015-04-08

**Authors:** Paul M. Ledger, Yannick Miras, Matthieu Poux, Pierre Yves Milcent

**Affiliations:** 1 Clermont Université, Université Blaise Pascal, Maison des Sciences de l'Homme, BP 10448, Clermont-Ferrand, France; 2 CNRS, USR 3550, MSH, Clermont-Ferrand, France; 3 CNRS, UMR 6042, GEOLAB, Clermont-Ferrand, France; 4 Department of Archaeology, University of Aberdeen, Aberdeen, United Kingdom; 5 Clermont Université, Université Blaise Pascal, GEOLAB, BP 10448, Clermont-Ferrand, France; 6 Université Lumiere Lyon 2 – ARAR UMR 5138 Archéologie et Archéométrie, Lyon, France; 7 Université de Toulouse – Jean Jaurès, UMR 5608-TRACES, Toulouse, France; University of Lausanne, SWITZERLAND

## Abstract

Early human societies and their interactions with the natural world have been extensively explored in palaeoenvironmental studies across Central and Western Europe. Yet, despite an extensive body of scholarship, there is little consideration of the environmental impacts of proto-historic urbanisation. Typically palaeoenvironmental studies of Bronze and Iron Age societies discuss human impact in terms of woodland clearance, landscape openness and evidence for agriculture. Although these features are clearly key indicators of human settlement, and characterise Neolithic and early to Middle Bronze Age impacts at Corent, they do not appear to represent defining features of a protohistoric urban environment. The Late Iron Age Gallic Oppidum of Corent is remarkable for the paucity of evidence for agriculture and strong representation of apophytes associated with disturbance. Increased floristic diversity – a phenomenon also observed in more recent urban environments – was also noted. The same, although somewhat more pronounced, patterns are noted for the Late Bronze Age and hint at the possibility of a nascent urban area. High percentages of pollen from non-native trees such as *Platanus*, *Castanea* and *Juglans* in the late Bronze Age and Gallic period also suggest trade and cultural exchange, notably with the Mediterranean world. Indeed, these findings question the validity of applying *Castanea* and *Juglans* as absolute chronological markers of Romanisation. These results clearly indicate the value of local-scale palaeoecological studies and their potential for tracing the phases in the emergence of a proto-historic urban environment.

## Introduction

The environmental impacts of the diffusion of agriculture and establishment of sedentary communities in Western and Central Europe can be traced through a variety of palaeoenvironmental archives [1]. From the Neolithic onwards, there is manifold evidence for the disruptive effect of agriculture on a wide range of natural process [2]. Forest clearance, the establishment of farming and the consequent development of anthropogenic cultural landscapes from c. 6000 cal. BP onwards has long been recognized in palaeoecology [3, 4, 5] while human impacts in other areas of the biosphere are also increasingly evident. Neolithic livestock husbandry, for example, has been implicated in eutrophication of water bodies [6] and the influence of land management practices in altering geomorphological regimes is well established [7]. Increasingly the impact of early agriculture is being viewed as more severe than previously acknowledged with some suggesting that the Neolithic revolution marks the beginning of the Anthropocene [1, 8].

Despite the plethora of studies documenting the landscape-scale disturbance of early farming activities in central Europe, there is often little direct consideration of the localised environmental impacts associated with the emergence of proto-urban and urban centres. Until recently this owed much to the predominant archaeological narrative that has tended to view societies north of the Alps as more tribal in nature [9]. Urbanism was considered a process limited to the Mediterranean that blossomed towards the end of the Iron Age and was accelerated by the Roman conquest [10]. Nevertheless, site selection bias in palaeoecology has also conspired to obfuscate the environmental impact of proto-urban areas. Much palaeoecological study has operated at the landscape-scale seeking to trace long term Holocene vegetation dynamics [11] and discern if climate or human activity is the primary driving force in landscape change (e.g. [12, 13, 14]). Frequently a ‘pristine’ environmental record, whereby the two signals can be easily disentangled, is considered essential in such studies. However, six millennia of human activity in lowland areas – such as the exploitation of peatlands for fuel, water abstraction and the drainage of wetlands – have led to the degradation, truncation, or destruction of lowland sediment archives within the milieu of human settlement. Even when human impacts are the focus of research, the taphonomic integrity of a palaeo-archive, rather than its signal sensitivity, is often considered the primary concern [15]. Consequently an abundance of palaeoecological studies are undertaken on deposits in more remote upland areas distant from the foci of proto-historic settlement (e.g. [16]). Such studies benefit from being relatively untouched by the taphonomic problems described above, but often lack the signal sensitivity to address, in any detail, questions pertaining to the localised environmental impacts of early human activity.

**Fig 1:**
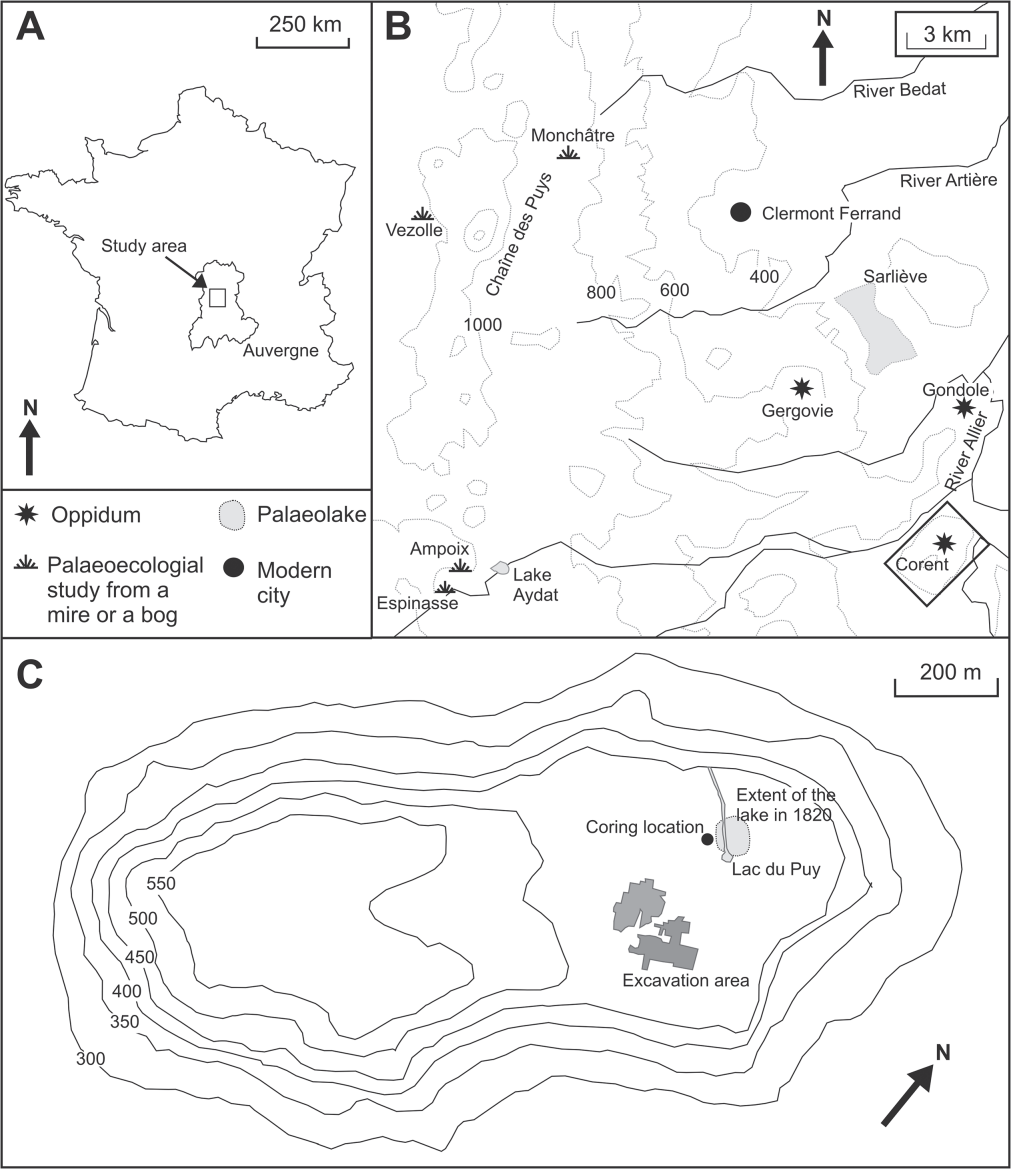
(A) Location of Auvergne and the study region within France; (B) Palaeoecological sites and Oppida surrounding Clermont-Ferrand; (C) The Plateau of Corent.

Auvergne in central France (Fig. 1) is a prime example of such bias in palaeoecological data. Moreover, it is an excellent location to evaluate, in the *longue durée*, the palaeoenvironmental impacts associated with human settlement, and the slow and chaotic development of urban areas. From the Neolithic onwards, there is abundant archaeological evidence for settlement in the valley of the River Allier, which intensified into the Bronze Age with the development of hill-forts [17] and culminated in a tripartite of large, interconnected fortified urban settlements (Oppida) in the late Iron Age [18]. Yet, there has been little direct consideration of the palaeovegetational impacts associated with the emergence of these proto-historic urban centres. The reasonably wide coverage of palaeoecological data for Auvergne is almost exclusively focused in mountainous areas and concerns the post-glacial vegetation evolution in the region (e.g. [19, 20, 21]). Only Miras et al [16] have directly considered long term human impacts, identifying evidence for cereal agriculture from c. 3000–2000 BC, but again this study derived from a mountainous area. Where lowland studies are available [22], the taphonomic problems mentioned above are prevalent and secure chronologies elusive [23, 24]. Thus, despite the existence of a rich history of archaeological and palaeoecological study, the dynamics of human-environment interactions in this part of central France are still poorly understood.

This paper aims to address these deficiencies by providing what we believe is the first palaeoecological study of a sediment core sourced from within a proto-historic settlement. The site, Puy de Corent (Fig. 1), was first occupied in the Middle Neolithic (c. 4000–3400 BC) and underwent waves of increasingly dense settlement from the Bronze Age onwards. These culminated in the Late La Tène with an urban Oppidum [18] which makes it an ideal location to examine the long term evolution of a proto-historic urban area. Utilising a sedimentary sequence from the Lac du Puy, located close to the heart of the ancient city, we apply palaeoecological techniques (pollen and non-pollen palynomorph analysis) and ^14^C dating to ask: (i) Is it possible to trace the various phases of human activity and the emergence of the Oppidum of Corent through the palaeoenvironmental record? (ii) What can palaeoenvironmental evidence tell us about the birth and development of an urban environment at Corent? (iii) How does the evidence differ, if at all, from previously published studies?

## Background

### Physical geography

**Fig 2:**
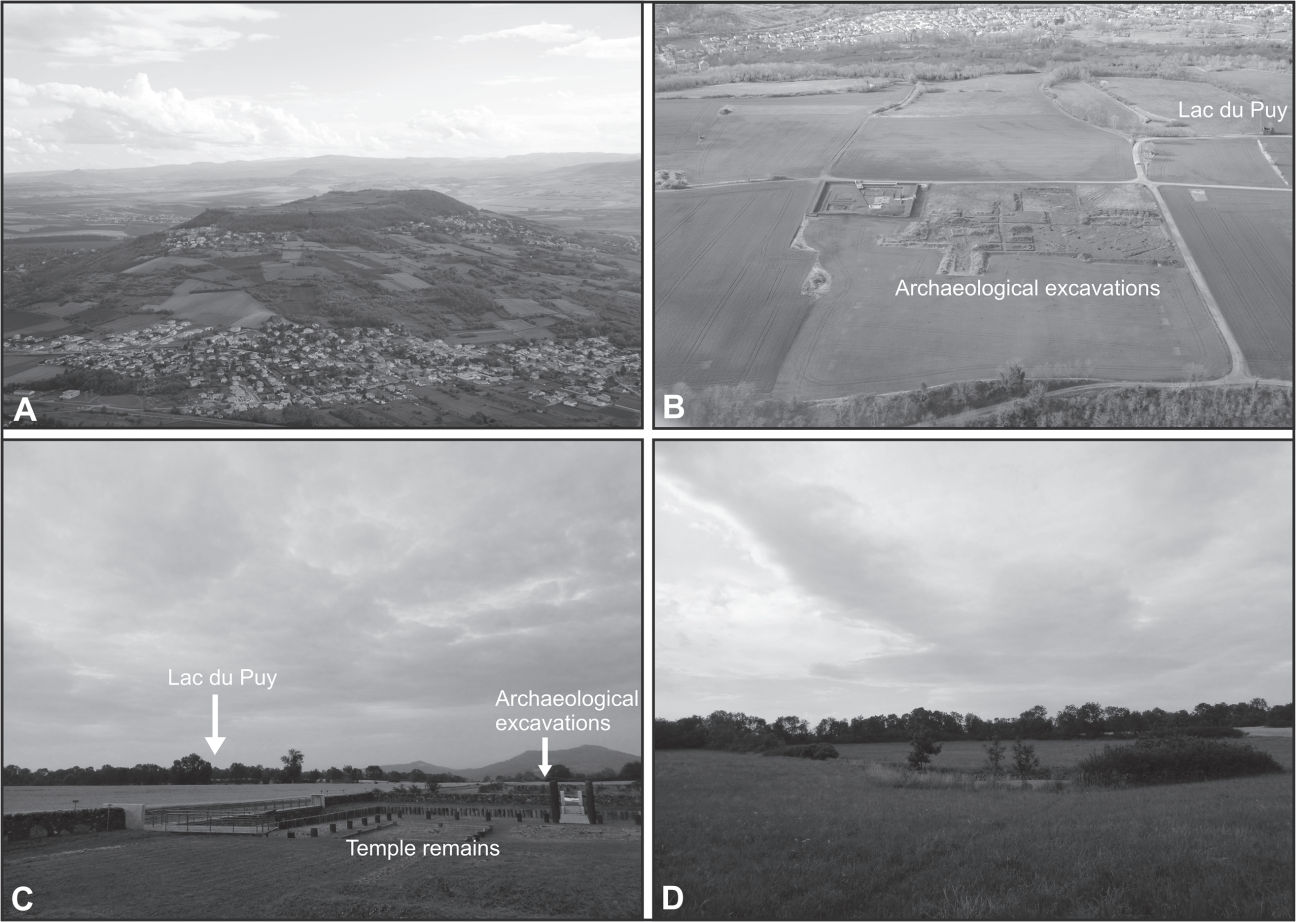
Photographs of the study site. [A] Corent plateau looking to the south (photo: B. Dousteyssier, 2014). [B] Aerial photograph looking northwest across the archaeological excavations and the Lac du Puy (photo: B. Dousteyssier, 2014). [C] View north across the plateau towards the Lac du Puy. Visible in the foreground are the excavated remains of a Gallo-Roman temple (photo: P.M. Ledger, July 2014). [D] View north across the Lac du Puy (photo: P.M. Ledger, July 2014).

The Corent plateau is located within the relict volcanic landscape of the Massif Central approximately 15 km southeast of Clermont-Ferrand at the heart of Auvergne in central France (Figs. 1, 2). At an altitude of between 500–600 m a.s.l. the plateau occupies a commanding position in the valley of the River Allier (c. 300 m a.s.l.) between the Chaîne des Puys to the west and the gradually undulating landscapes to the east. Climatically the region is continental with limited precipitation (585 mm yr^−1^) and experiences relatively cold winters (January mean 7.3°C) and warm summers (July mean 25.9°C). The geology of the plateau is uncomplicated and dominated by basalt to the north and east, and scoria to the south and west reflecting the plateau’s volcanic origin c. 3 Ma yrs BP [25].

### Archaeology

Auvergne is well known in the study of proto-historic or proto-urban centres (often termed Oppida) in Central and Western Europe. Some of the earliest archaeological study of these early urban centres outside of the Mediterranean was undertaken in Auvergne with the identification of the Oppidum of Gergovie immediately to the south of Clermont-Ferrand by Napoleon III in 1865 [9]. In contrast, Corent was, until relatively recently, somewhat unknown. Bronze objects, pottery, and coins were commonly found in surface soils on the plateau, leading to the suspicion that the plateau was of importance in Prehistory, but it was not until reappraisal in the early 1990s [26], and the beginning of annual excavations by Poux [27] that the sites importance came into focus. Since then annual field campaigns have revealed an immense, wealthy Oppidum – dating to the La Tène D1/2 (125–25 BC) – that contained public, commercial, artisanal and religious buildings. Moreover the rich material culture, indicating trade and contact with the *Mediterranean* world, led Poux [18] to suggest that the site may have functioned as the capital of the Arverni. Yet, the excavations have also demonstrated that the La Tène Oppidum is merely one short lived phase of occupation on the plateau. There is also evidence of large settlements in the Hallstatt (600–550 BC) and late Bronze Age (c. 950–800 BC), as well as discontinuous settlements that extend back to the Middle Neolithic [28].

## Methodology

### Fieldwork and sediment sampling

The Lac du Puy is a spring fed pool located in the northern quarter of the Corent plateau approximately 170 m north of the excavation area (Figs. 1, 2) centered at 45° 40’0.66” N. The open water area of the modern pool, measuring c. 20 x 20 m, is almost certainly of anthropogenic origin and is much reduced from the c. 65 x 72 m (0.46 ha) recorded in a map dating from 1820 (Fig. 1). This reduction in size is likely the result of agricultural improvements and the installation of a drainage channel in the Napoleonic era [18] and subsequent sediment infilling. Despite this evidence for management of the pool in the 19^th^ century, the basin is almost certainly of natural origin. The palaeobasin of the pool represents a depression within the impermeable basaltic bedrock which is at its deepest (c. 3 m) in the centre, and shallows towards the edges. Similar formations are common to neighbouring volcanic plateaux (e.g. Puy de Saint Sandoux). On the basis of the modern topography of the surrounding palaeobasin, it is probable that the open water area may have been as a large as c. 100 x 80 m (c. 0.8 ha). Modelling studies from other forested cultural landscapes in Europe suggest that RSAPs for similar sized basins would have been in the order of 1200–2300 m (c.f. [29]). This would suggest that the Lac du Puy was likely to have been dominated by local to extra local input, despite the setting, at the summit of the plateau, favouring the input of regional pollen. Nevertheless, it is important to note that RSAPs are dynamic and vary as the basin infills and the surrounding landscape mosaic changes.

In November 2012 judgmental coring of the palaeobasin of the lake was undertaken to identify the depth of organic deposits that are believed to have been unaffected by historical management. This proved organic deposits to a maximum depth of 186 cm. These were sampled (co-ordinates: 3° 11’ 21.30”E) in two overlapping 100 cm sections using a dynamic coring rig (Geotool GTR 790). The study was undertaken on private land and permission was provided by the landowner Madame Mioche. Core sections were protected in plastic tubes and wrapped in polythene before being returned to the Université Blaise Pascal where they were placed in cold storage (4°C). Sub-sampling, at 1 cm intervals (where possible), and recording of the lithostratigraphy was undertaken in the laboratory.

### Pollen analysis

Subsamples of between 1 and 3 cm^3^ were prepared for pollen analysis using standard HCl, sieving, HF, acetolysis and density separation techniques (solution density 1.9) outlined in Moore et al. [30]. *Lycopodium* tablets [31] were added to allow the calculation of pollen and microscopic charcoal concentration and influx data. After processing, the samples were suspended in silicone oil prior to being mounted on slides. Counting was undertaken using a Leica DM 2500M light microscope at x500 magnification (with x1000 used for critical observations) until a sum of 500 total land pollen (TLP) had been achieved. Pollen and spores were identified with the aid of the key in Moore et al. [30], photographs in Reille [32] and modern reference material, with nomenclature following Beug [33]. Cereal-type pollen grains were categorised following measurements in Andersen [34] and non-pollen palynomorphs (NPPs) were identified using van Geel et al. [35] and van Geel and Aproot [36]. Pollen diagrams were constructed using TILIA and TGView software [37] and percentages were based upon the TLP sum minus Lactuceae (see below for justification).

### Archaeological samples

In addition to the sedimentary sequence a total of thirty soil samples from archaeological contexts (each from different periods) were also processed for pollen (Table 1). This was undertaken with the intention of integrating pollen-analytical evidence from two different scales of analysis. In each instance approximately 5 cm^3^ of soil was prepared and examined using the methods described above.

**Table 1:** Summary of the archaeological sediments examined for pollen.

**Sample name**	**Archaeological period and approximate age**	**Archaeological context**
UF-23087	Roman (1–2^nd^ century AD)	Cellar perhaps from a pottery workshop.
UF-23124	Roman (between 15 BC and AD 15)	Interpreted as originating from a pottery workshop
UF-18242	Middle of the first century BC	Large deep cellar sealed by demolition rubble. Deposit contained numerous intact pots and ceramic fragments.
UF-15444	La Tène (end of 2^nd^ beginning of 1^st^ century BC)	Pit or post hole
UF-22839	Middle Hallstatt	Unspecified pit.

### Charcoal analysis

Microscopic charcoal concentrations were estimated utilising the point count method [38]. A total of 8800 points were applied to achieve a <5% error in charcoal estimates and concentrations are expressed as cm^2^ cm^−3^.

### Numerical analysis

**Fig 3:**
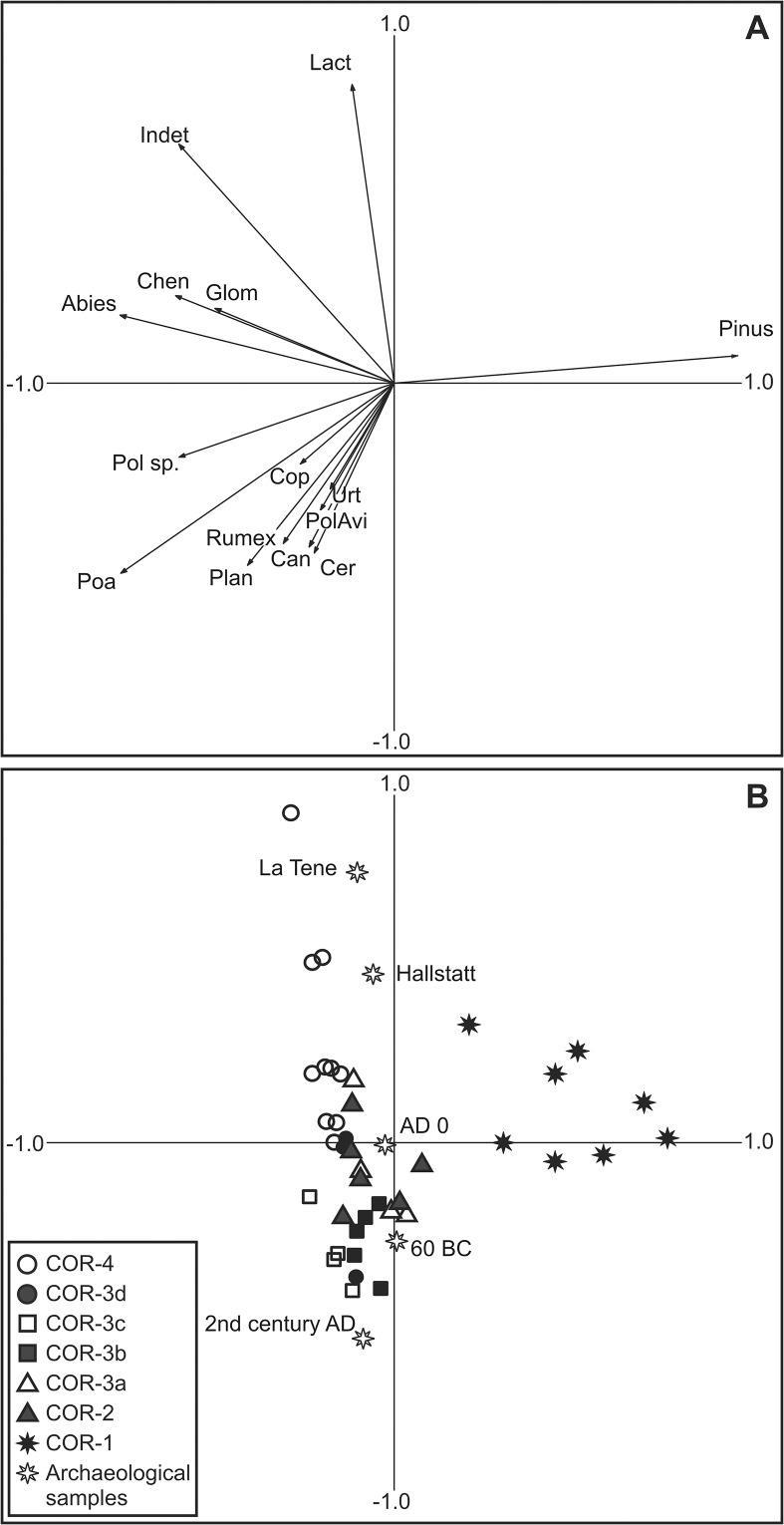
PCA plots for the profile. A. Pollen types and associated proxies (selected taxa only). B. Sample scores for pollen and spore dataset with archaeological samples included as passive samples. Key to abbreviations: Can = Cannabis/Humulus; Cer = *Cerastium*-type; Chen = Chenopodiaceae; Cop = coprophilous fungi; Glom = *Glomus*; Indet = Indeterminable pollen; Lact = Lactuceae; Plan = *Plantago* sp.; Poa = Poaceae; PolAvi = *Polygonum aviculare*-type; Pol sp. = *Polygonum* sp.; Urt = Urticaceae.

CONISS [39] was used to assist with the biostratigraphic zonation of the pollen diagram following square root transformation of percentage data. Rarefaction analysis was performed in *psimpoll* [40]. This was used to determine the palynological richness, and hence floristic diversity, of samples [41]. Ordination of pollen samples was undertaken using CANOCO 4.5 [42]. In the first instance detrended correspondence analysis (DCA) was undertaken which generated a gradient length of 1.685 for the primary axis. This indicated a linear rather than unimodal response in the dataset [43] and principal components analysis (PCA) was therefore judged to be more appropriate (Fig. 3). Pollen samples from archaeological contexts were included in the PCA ordination as passive (supplementary) samples in order to examine their relationship with samples from the sediment core.

### Radiocarbon dating and age-depth modelling

AMS ^14^C dating was undertaken on charcoal, pollen and the bulk organic fraction (BOF) of sediment samples (Table 2). In the first instance blocks of sediment were cut from the core and placed in Sodium hexametaphosphate solution in order to disaggregate. After 24 hours the samples were washed through 200 μm and 125 μm sieves with distilled water and examined under a low power binocular microscope for charcoal and macrofossils. Unfortunately, suitable macrofossils were absent and sufficient charcoal was identified in only one instance, between 57–54 cm. This sample was selected along with a sediment sample from the same depth in order to assess the potential reservoir effect associated with the BOF. Similarly, pollen was concentrated from between 73–71 cm following the method outlined by Brown et al [44] and submitted for ‘paired’ dating with a sediment sample from 72–71 cm. In addition two further samples of sediment were submitted for BOF dating. Samples were pre-treated and measured at Beta Analytic, Miami, USA. Calibration of ^14^C dates was undertaken using the IntCal13 calibration curve [45] and CALIB v7.0. Age-depth modelling was performed using CLAM version 2.2 [46].

**Table 2:** Radiocarbon dates from the profile.

**Depth (cm)**	**Lab. code**	**Material**	^14^ **C yr BP (±1σ)**	**Cal BC/AD (2 σ)**	**δ** ^13^ **C (‰)**
57–54	Beta-379416	Charcoal	2240 ± 30	390–205 BC	NP
57–54	Beta-379417	Bulk sediment	1990 ± 30	48 BC – AD 71	−25.6
72–71	Beta-377232	Bulk sediment	2590 ± 30	819–595 BC	−26.4
73–71	Beta-379418	Pollen	2750 ± 30	975–823 BC	NP
88–87	Beta-375785	Bulk sediment	3510 ± 30	1916–1749 BC	−25.4
103–102	Beta-379419	Bulk sediment	3330 ± 30	1688–1528 BC	−25.7

## Results

### Lithostratigraphy

The cored sequence can be broadly divided into four main units (Table 3). From the base, at 186 cm, the sediment comprises scoria, sand and gravel, which at 163 cm becomes slightly clayey. The most significant change occurs at 118 cm where there the sediment becomes grey brown sandy silty clay and is clearly lacustrine. Pottery fragments are evident through this unit from c. 100 cm and particularly abundant from c. 85 cm. From 48 cm there is a shift towards brown silty clay which is highly disturbed, perhaps associated with more recent farming activity or management.

**Table 3:** Lithostratigraphy of the core.

**Depths**	**Unit description and Troels-smith formula**
48–0	Highly disturbed brown slightly sandy silty clay containing frequent rootlets and occasional monocot remains. Basalt and flint gravels are occasional. Pottery fragments are abundant throughout. As2 Ag1 Gmin1 Th^4^+ Ga+ Nig 3 Sicc 1–2 Strf 0 Lim 1
118–48	Grey brown silty clay with occasional basaltic and scoria gravel and rare plant remains including monocot stems and unidentified rootlets and seeds. Small pottery fragments are noted from c. 100 cm and particularly abundant from c. 85 cm. As3 Ag1 Ld++ Gmin ++ Ga++ Th^4^++ Nig 2–3 Sicc 1–2 Strf 2 Elas 3 Lim 1
163–118	Brown mottled red orange and yellow clayey sand and gravel. Gravel comprises basalt and scoria. Gmin2 Ga1 As1 Ag+ Nig 3 Sicc 3 Strf 2 Elas 0 Lim 0
186–163	Red brown slightly sandy gravel comprising abundant scoria. Ga2 Gmin2 Ag+ Nig 3 Sicc 3–4 Strf 0 Elas 0 Lim NA

### Statistical analysis

PCA indicated an excellent separation of dataset with the primary two axes accounting for 76.3% of the variance (Fig. 3). The primary axis (61.6%) is dominated by a strong positive response in *Pinus* and a negative response in Poaceae. Although this gradient – contrasting *Pinus* with Poaceae – initially appears to simply reflect landscape openness and human impact the presence of negative responses in other tree species such as *Abies* suggests a more nuanced interpretation. The association of *Abies* – which is more common to mountainous environments in Auvergne [16] – with Poaceae perhaps, indicates that the axis also reflects changing RSAP and recruitment of pollen from a wider area. This is not unexpected since models demonstrate that the opening of a landscape around a basin will increase its RSAP [29].

Axis 2 (14.7%) is characterised by positive loadings for Lactuceae and indeterminable pollen and slightly negative responses for cereals and apophytes (e.g. *Rumex* and *Plantago* sp.). This pattern does not immediately suggest an obvious ecological gradient. Lactuceae pollen – with a strong resistant exine – and indeterminable pollen have been found to be enriched in deposits where preservation conditions are poor [47]. This association of Indeterminable and Lactuceae pollen therefore suggests that axis 2 may reflect the degree to which samples are affected by post depositional biasing, either by means of *in-situ* decay or the input of secondary pollen. Assuming that an element of the high numbers of Lactuceae reflect secondary pollen, rather than contemporary vegetation communities, the taxon has been excluded from the TLP sum used to calculate pollen percentages.

### Chronology

The results of radiocarbon dating are presented in Table 2. The clearest observation from the data is that measurements undertaken on paired charcoal/microfossil-BOF samples presented no evidence for a reservoir effect in the BOF dates. Microscopic charcoal from 57–54 cm (Beta-379416) returned a date of 2240±30 which was 250 radiocarbon years older than the date on the BOF at the same level (Beta-379417; 1990±30) suggesting a c. 200 cal. yr difference in the age of this level (Table 4). A similar, although less pronounced, discrepancy was observed in the paired dating from 73–71 cm. Pollen from this depth (Beta-379418) dated to 2750±30 whereas the BOF (Beta-377232) indicated an age of 2590±30. These results perhaps indicate the erosion and reworking of previously deposited material from the surrounding basin, suggesting that contrary to expectations, BOF dates may be preferable to highly mobile microfossils in this depositional environment. Nevertheless, this is far from certain as the final two dates on BOF at 88–87 cm (Beta-375785; 3510±30) and 103–102 (Beta-379419; 3330±30) present a minor age-depth reversal hinting at the possibility of a reservoir effect.

**Table 4:** Comparison of the two age-depth models generated in Fig. 4. Age ranges in the table are cal. BC. Numbers in parentheses reflect the median age from each model.

**LPAZ**	**Depth (cm)**	**Linear regression**		**Smoothed spline**	
		**Age (2σ)**	**Period**	**Age (2σ)**	**Period**
COR-4	69.5	775–640 (705)	Early Hallstatt	815–700 (745)	Early Hallstatt
COR-3d	75.5	1015–865 (935)	Late Bronze	1075–920 (990)	Late Bronze
COR-3c	83.5	1335–1170 (1250)	Late Bronze	1415–1180 (1290)	Middle/Late Bronze
COR-3b	93.5	1735–1545 (1640)	Middle/Late Bronze	1870–1465 (1610)	Early/Middle Bronze
COR-3a	101.5	2055–1850 (1950)	Early Bronze	2225–1675 (1830)	Early Bronze
COR-2b	110.5	2420–2190 (2300)	Late Neolithic	2625–1875 (2065)	Late Neolithic
COR-2a	122.5	2905–2645 (2770)	Late Neolithic	3160–2090 (2385)	Late Neolithic
COR-1b	145	3800–3495 (3645)	Late/Middle Neolithic	4155–2470 (2985)	Late/Middle Neolithic
COR-1a	152	4080–3760 (3920)	Late/Middle Neolithic	4455–2560 (3170)	Late/Middle Neolithic

**Fig 4:**
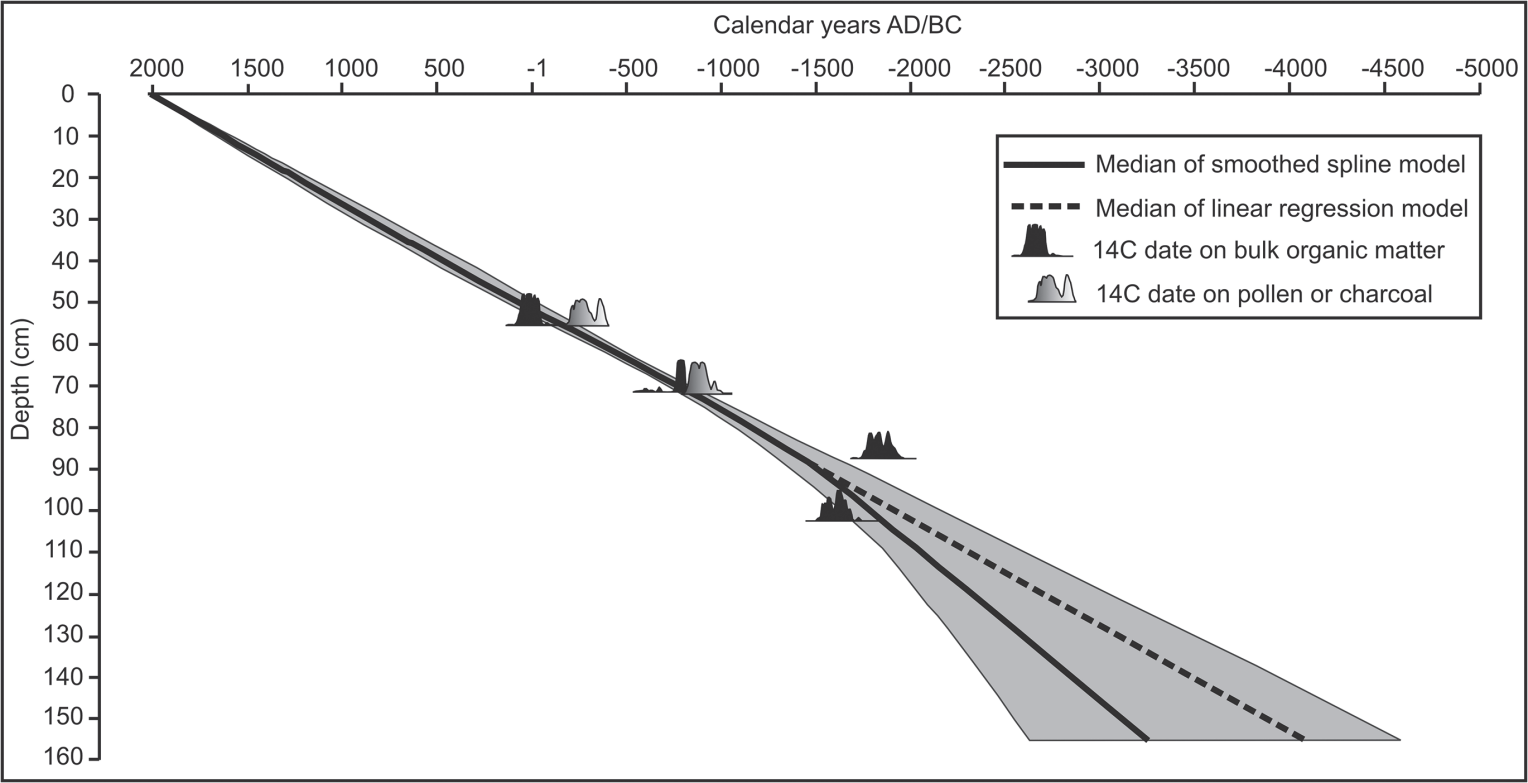
Age-depth model for the sequence generated using Clam (Blaauw, 2010). Two possible models, generated using a smoothed spline and linear regression, are presented. The grey envelope indicates the 95% confidence limits of the smoothed spline model which encompasses the narrower 95% confidence limits of the linear regression model. The solid black line and dashed line respectively represent the ‘best estimates’ of the smoothed spline and linear regression models.

All of the radiocarbon data was used to construct the age-depth model (Fig. 4), a process deemed to be preferable to excluding samples on the basis of supposition. Two methods, linear regression and smoothed splines (polynomial regression), were explored and in both instances the models were extrapolated to the base of the analysed sequence at 155 cm. There is little difference between the linear regression and smoothed spline models for the majority of the sequence (Table 4), however, below c. 95 cm the two models diverge and provide substantially different results for the basal portion of the analysed sequence. In the absence of dating control for the basal portion of the sequence we have adopted the chronology of the smoothed spline model which better reflects the uncertainty surrounding the age of this section of the core. All dates relating to palynological data that are presented in subsequent sections are two sigma modeled age ranges of the smoothed spline model. Where events in pollen record have been assigned a single date, this reflects the best fit age from the model.

### Palynology – archaeological samples

**Fig 5:**
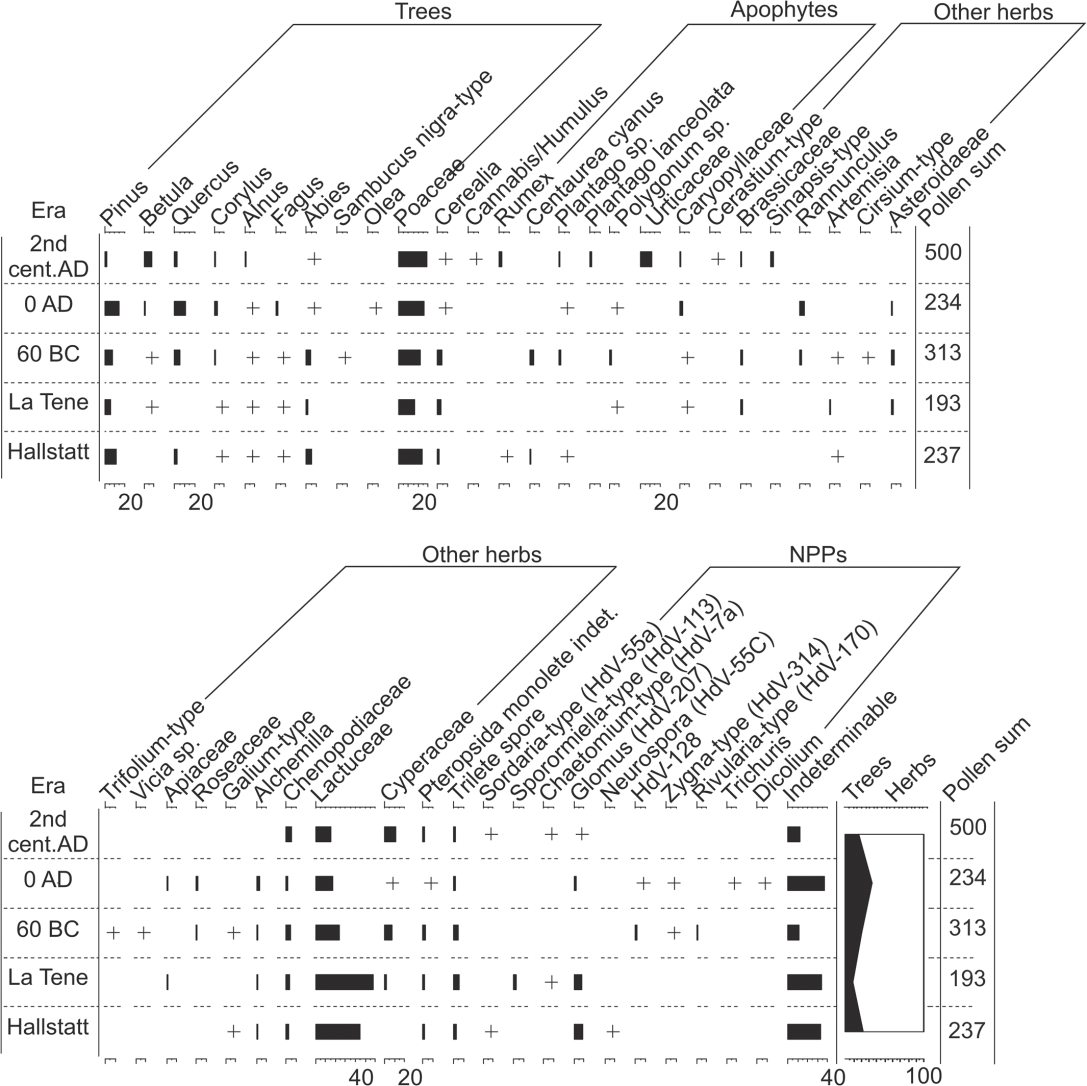
Percentage pollen and spore diagram for archaeological samples, displaying selected taxa (based on the TLP sum). Also shown is the summary diagram and pollen sum for each sample. + indicates <1% TLP.

In total only five sediment samples from varying archaeological contexts and eras contained sufficient pollen to undertake counts (Table 1; Fig. 5). The clearest observation from this data is the general lack of inter sample variation in the pollen assemblages and that they compare favourably to similar analyses from sites in Auvergne (e.g. [23]). Typically arboreal pollen accounts for between 10–20% of the assemblage with *Pinus*, *Quercus*, *Betula*, *Corylus* and *Abies* being the dominant types (Fig. 5). Poaceae percentages range between 17–29%, cereal pollen is present in each sample and the assemblage of herbs is broadly comparable. Only UF-23807, dating to the 2^nd^ century AD, differs slightly with higher percentages of apophytes (mainly Urticaceae and *Rumex*) and a less diverse assemblage of herbs. Thus, the clearest difference between the samples is in their inferred preservation state. Older samples tend to record positive scores along axis 2 of the PCA and younger samples generally respond negatively (Fig. 4). This is apparent in the low pollen sums, high Lactuceae counts of between 45–55% and Indeterminable pollen percentages in excess of 30% for samples dating to the La Tène and Hallstatt periods (Fig. 5).

### Palynology—sediment core

**Fig 6:**
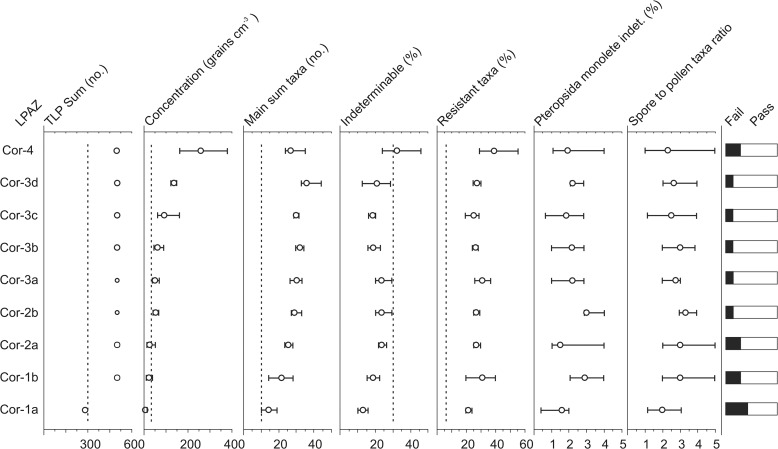
Assessment of the taphonomy of the core based on tests proposed by Bunting and Tipping (2000). Circles reflect the mean values with error bars indicating the maximum and minimum values for each LPAZ. Dashed lines indicate the tresholds below which samples fail each test expect in the case of Indeterminable percentages where values greater than the threshold indicate failure.

**Fig 7:**
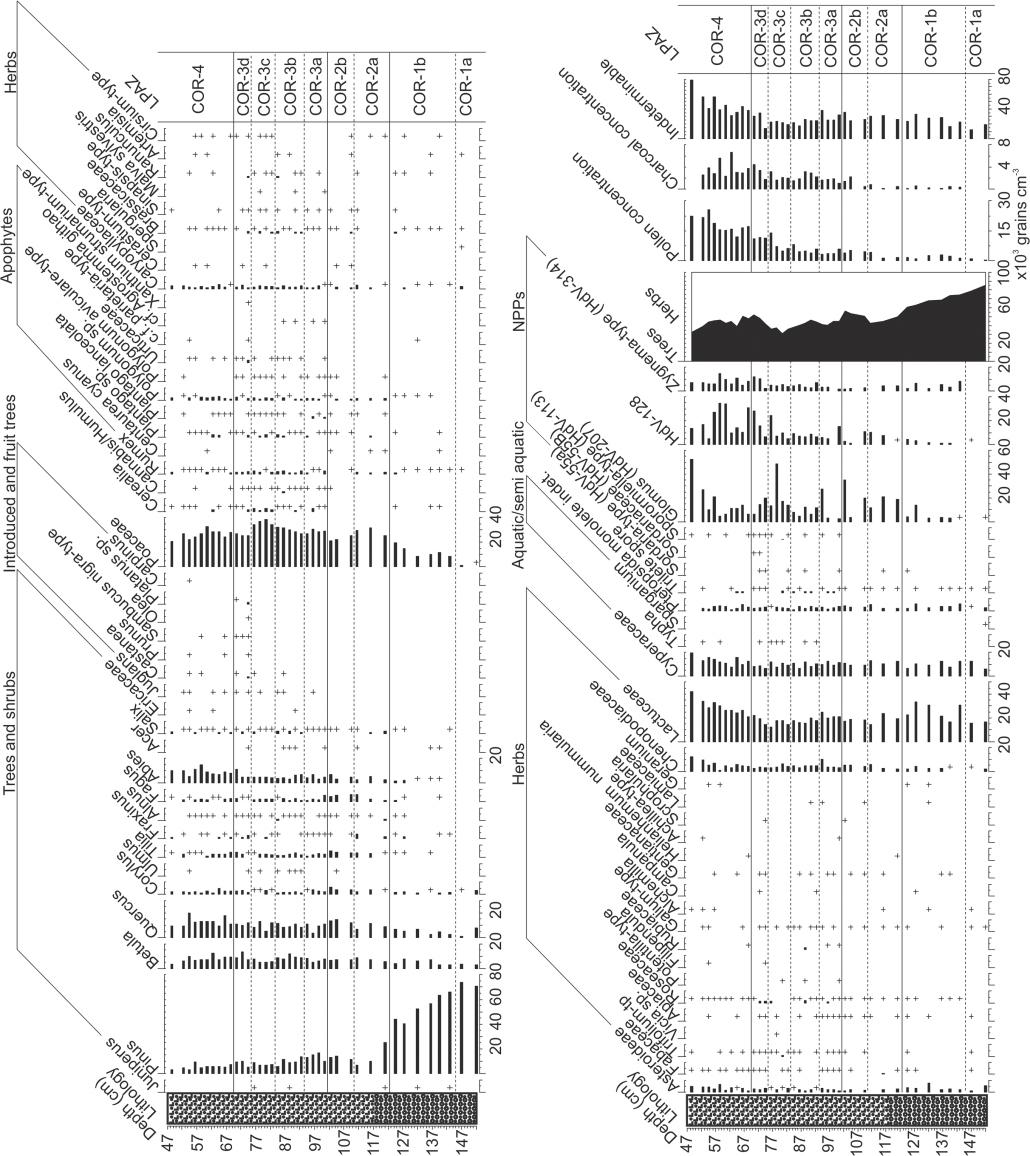
Percentage pollen and spore diagram displaying selected taxa (based on TLP sum – Lactuceae). Also shown is the lithology, microscopic charcoal and total pollen concentration. + indicates <1% TLP.

Given the provenance of the core from the palaeo-basin of the Lac du Puy, evidence for historical management and a lithology which does not indicate ideal conditions for the preservation of pollen it is prudent to assess the taphonomic integrity of the pollen data. Bunting and Tipping [47] suggested eight tests to ascertain evidence for taphonomic problems, which have been applied to the palynological data on a zone by zone basis (Fig. 6). The clearest finding of this analysis is that each zone easily exceeds the suggested 6% TLP ‘resistant taxa’ TLP threshold. Primarily this is a result of high Lactuceae pollen percentages which are ≥15% TLP (Fig. 7) through each local pollen assemblage zone (LPAZ) of the core. The consistency of this pattern – even through zones which otherwise do not indicate taphonomic problems (Figs. 6, 7) – suggests that these elevated percentages do not necessarily indicate poor preservation of pollen. Rather it may be the case that Lactuceae pollen reflects the erosion and deposition of ‘old’ secondary pollen from soils in the surrounding catchment [48]. Furthermore, the majority of the pollen types in the resistant taxa category derive from species which are known to respond to human activity. The utility of the ‘resistant taxa’ measure, in the context of a human impact study, is therefore debatable and failure of this test is not considered to have serious implications for the interpretation of the assemblages that only fail this test. More concerning are the assemblages from the basal three zones (COR-1a, 1b and 2a). Mean pollen concentrations are lower than the 3000 grains cm^-3^ threshold recommended by Bunting and Tipping [47] while each TLP sum in COR-1a was below 300 grains. These results indicate post depositional pollen loss and that data from the basal three zones should be interpreted with caution. The same conclusion applies to LPAZ COR-4 where indeterminable pollen percentages frequently exceed the recommended 30% threshold (Fig. 6).

The opening of COR-1a dates to 4455–2560 cal. BC, which is congruent with the presence of arboreal pollen (AP) characteristic of the Atlantic [19]. *Pinus* (70–75%) dominates and there are smaller contributions from *Betula*, *Quercus* and *Corylus* (Fig. 7). Although AP accounts for 75–85% TLP in this zone, it is not necessarily indicative of especially dense forest on the summit of the plateau. *Pinus*, the dominant taxon, is a high pollen producer [30] and these values may simply reflect local Pine woodland around the depositional basin. Indeed, low pollen concentrations imply significant post-depositional pollen loss [47] that may have resulted in an enriched representation of *Pinus* pollen relative to other species.

COR-1b is characterised by the first evidence of human activity on the plateau. Microscopic charcoal concentrations increase, the AP contribution continues to decline and there is a rise in Poaceae to c. 12% that indicates the gradual opening of the landscape. The age-depth model (Fig. 4) suggests the beginning of this zone dates to the Middle to Late Neolithic (4155–2470 cal. BC). This is in general agreement with the first appearances of taxa such as *Fagus* and *Abies*, and is consistent with indications of limited human impact. Cereal pollen is only rarely present in COR-1b and the sole other indicator of agriculture is a single grain of *Centaurea cyanus*. Considering the small size of the depositional basin and indications of relatively dense surrounding woodland, it is likely these values indicate local activity [30]. Aside from the evidence for cultivation, there are signs of trampling and general disturbance of the vegetation. *Rumex* is frequently present in COR-1b and pollen from *Plantago* sp. and *Polygonum* sp conceivably reflect *Plantago lanceolata* and *Polygonum aviculare*, plants that are common to a variety of ruderal environments across France [49]. A general diversification of the herb flora, with regular representation from Caryophyllaceae and Brassicaceae, may also be related to disturbance and possibly grazing [50]. However, the absence of coprophilous fungal spores suggests that general disturbance is the more likely explanation.

The beginning of COR-2a dates from the Late Neolithic (3160–2090 cal. BC) and represents a period of major forest clearance on the plateau. *Pinus* falls from 45% to 7% by the end of the zone and, despite the emergence of species such as *Abies* (3–6%), *Fagus* (1–6%), *Tilia* (1–4%), *Fraxinus* (1–2%) and increased representation of *Quercus*, there is an overall decline in AP from 61 to 42% (Fig. 7). Whether the rise in *Fagus* and *Tilia* reflect beech and lime woodland on the Corent plateau, or communities along the slopes is unclear. *Tilia* and *Fagus* are more commonly associated with lower altitudes in Auvergne, but the species can be found at up to 900 m a.s.l. [51] and it is therefore possible these values reflect isolated stands of these species within wider Oak and Pine woodland. Human agency is almost certainly responsible for the opening of the landscape recorded in COR-2a. Microscopic charcoal concentrations rise and are concurrent with a sharp increase in Poaceae. Evidence for landscape disturbance is also clear with a peak in *Glomus* suggesting erosion of catchment soils [52]. Traces of the apophytes *P*. *lanceolata* and *P*. *aviculare*-type as well as consistent representation from probable apophytes such as *Rumex* (1–2%) and *Polygonum* sp (1%) again imply the presence of trampled areas, yet evidence for agriculture is limited to occasional grains of *C*. *cyanus*. Cereal-type pollen disappears from the assemblage in COR-2a and there is only a single *Sordaria*-type spore that is suggestive of grazing animals [35].

COR-2b (beginning 2625–1875 cal. BC) marks a further significant change in the history of the plateau. There is a strong recovery in AP percentages to c. 57% (predominately *Pinus* and *Quercus*) and a fall in Poaceae to 21%, implying the regeneration of local Pine and Oak woodland. *Tilia*, *Fagus* and *Abies* remain almost static (Fig. 7), suggesting these species reflect the extra-local to regional vegetation rather than woodland local to the plateau. A reduction in pollen from apophytes appears to suggest these developments are related to a decline in human activity. Nevertheless, traces of *Plantago* sp., *P*. *aviculare*, *P*. *lanceolata* pollen and elevated microscopic charcoal concentrations are not suggestive of total abandonment.

**Fig 8:**
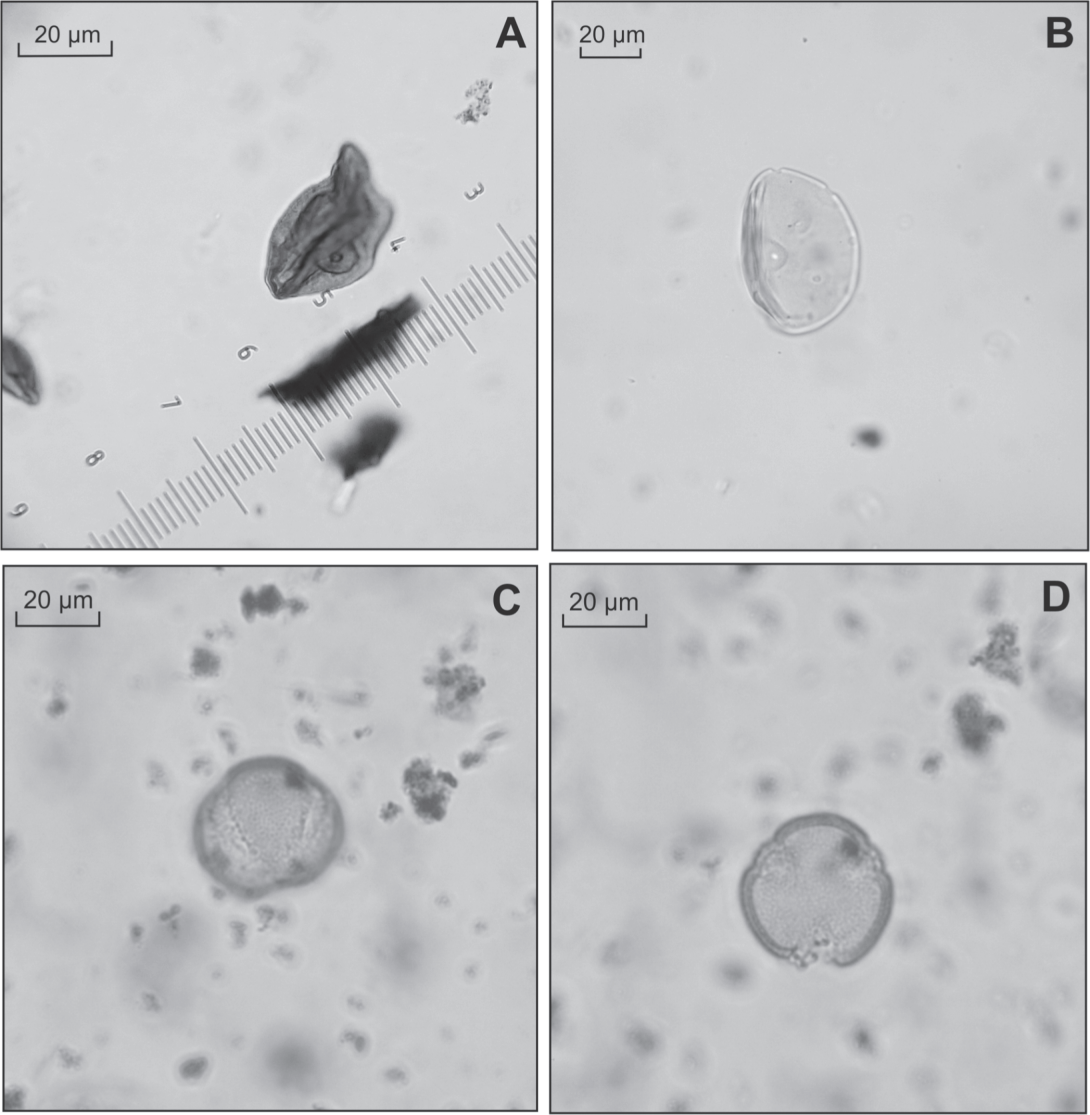
Images of pollen types identified in the core. (A) Highly folded cereal pollen (probably *Hordeum*-type) from 140–139 cm with a mean size of 39 μm, annulus of 8.5 μm and pore of 3 μm; (B) *Juglans* pollen identified at 97–96 cm; (C) *Platanus* (equatorial view) from 75–74 cm; (D) *Platanus* (polar view).

The opening of COR-3a, dating to 2225–1675 cal. BC (Early Bronze Age), marks the beginning of a second significant episode of woodland clearance. AP falls to a minimum of 41%, primarily a result of a declining contribution from *Quercus* and *Corylus*, and to a lesser extent *Fagus* (Fig. 7). Other tree species, notably *Betula*, *Tilia* and *Fraxinus*, remain relatively constant while *Pinus* records a slight increase from the previous zone. This bias, towards taxa more likely to be present on the plateau suggests the developments of COR-3a reflect highly localised vegetation changes. Moreover, the concurrent expansion of Poaceae, significant increase in pollen from apophytes and sharp rise in microscopic charcoal all point to a period of renewed human activity. Cereal percentages that reach a maximum of 2% and pollen from weeds common to fields such as *Agrostemma githago* (corn cockle), and to lesser extent *P*. *lanceolata*, suggest arable agriculture [49]. A further development is the first registration of the coprophilous fungi *Sporormiella*-type (common to the dung of grazing herbivores), which may even imply some pastoral activity [35]. Significantly COR-3a also marks the first appearance of pollen from *Juglans* (walnut) dating to c. cal. 2000–1545 BC (Figs. 7, 8). In the Italian peninsula and the eastern Mediterranean, *Juglans*, dating to the early Bronze Age, is common and considered an indicator of agricultural expansion in pollen diagrams [53]. Yet, its introduction to France is typically associated with the Roman conquest and process of Romanisation [11].

A minor recovery of woodland is suggested from the beginning of COR-3b (1870–1465 cal. BC) with a slight increase in AP, most notably *Tilia* and *Betula* (Fig. 7). This, however, is not sustained and AP percentages fall to a low of 38% by the end of the zone, concurrent with a rise in Poaceae to 32%. These developments point to further opening of the landscape and the regularity of cereal pollen indicates an increase in the area under cultivation. These observations are consistent with the continued presence of apophytes indicative of disturbance and cultivation. Again, as in COR-3a, there is little evidence of pastoral agriculture or grazing animals, with only a rare presence of coprophilous fungi such as *Sordaria*-type and *Sporormiella*-type. Further occurrences of *Juglans* are also noted, as is the first appearance of *Castanea* (chesnut) dating to 1550–1270 cal. BC.

The opening of LPAZ COR-3c dating to 1415–1180 cal. BC (the beginning of the Late Bronze Age) marks a significant increase in evidence for human impact. Poaceae peaks at 38% and AP falls to 31%, the lowest value in the core, predominately as a result of declines in *Pinus*, *Betula* and *Quercus* (Fig. 7). Given the localised RSAP of the depositional basin it is likely that these values indicate a very open landscape on the summit of the plateau, with woodland likely confined to the slopes and margins. The stagnant curves for *Abies*, *Tilia* and *Fagus* would appear to confirm that the decline in AP is related to local woodland clearance on the plateau. Concurrent with this reduction in woodland is an expansion in evidence for agriculture as Cereal-type pollen reaches maximal values of between 2–3%. *Plantago* and *Polygonum* pollen is also very well represented and likely derives from weeds such as *P*. *lanceolata* and *P*. *aviculare*. Traces of *Sporormiella*-type throughout the zone hint at the possibility of pastoral activity.

COR-3d (opening 1075–920 cal. BC) is characterised by increased AP and falling Poaceae pollen percentages that suggest a reduction in the intensity of human impact. Evidence for agricultural activity also declines as Cereal-type pollen is reduced to trace amounts. There is, however, evidence for possible pastoral activity with consistent representation of *Sporormiella*-type and the presence of other coprophilous fungi such as *Sordaria*-type and Sordariaceae. A further clear development in COR-3d is the diversification of the pollen assemblage. Pollen from a number of fruit trees such as *Prunus*, *Sambucus nigra*-type and *Olea* are first recorded and *Castanea* reaches 1%. In addition, pollen from *Platanus* (plane trees) is recorded at up to 1% (Figs. 7, 8).

**Fig 9:**
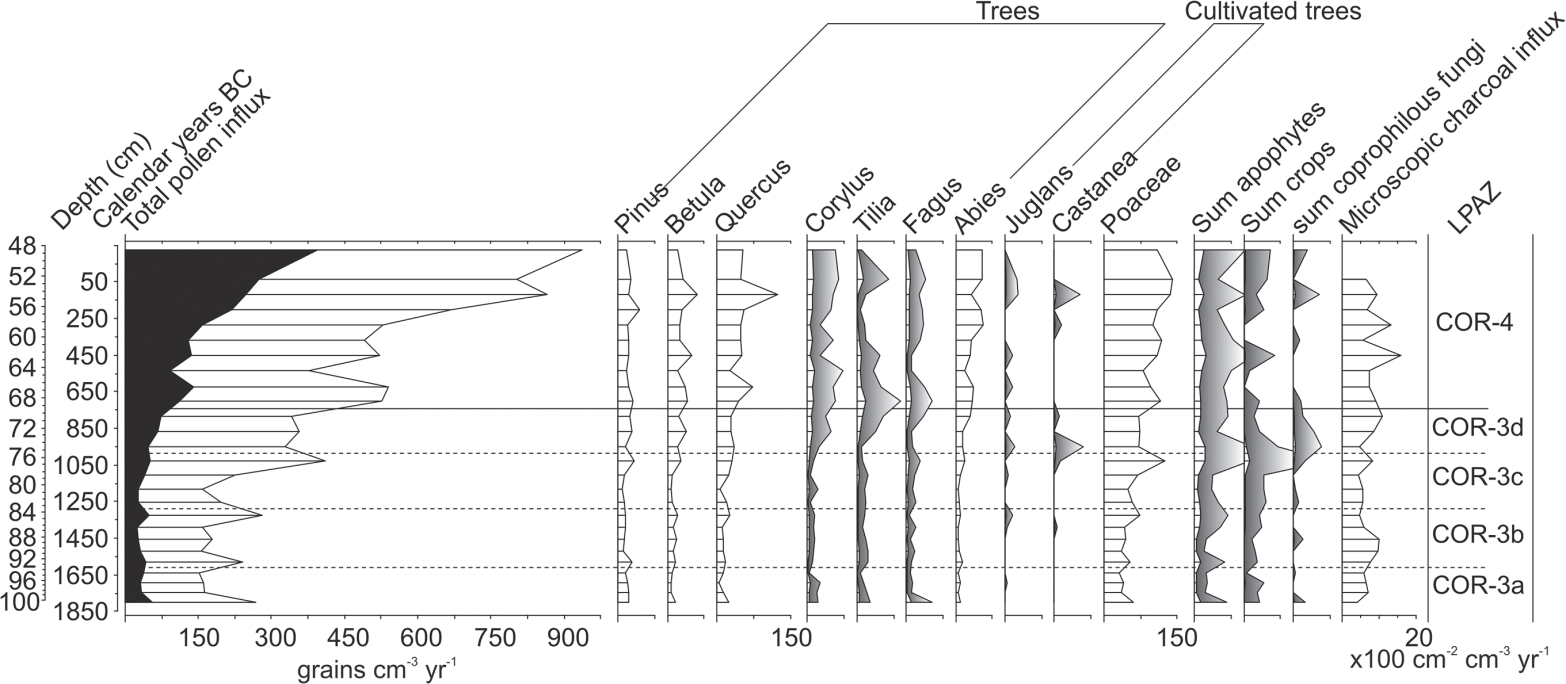
Pollen influx diagram showing total pollen influx with the Lactuceae curve overlaid (black), selected pollen types, sum of apophytes, sum of crops, sum of coprophilous fungi and microscopic charcoal influx plotted against the best fit from the smoothed spline Clam model. Exaggeration curves are x 5 for tree taxa and x10 for others. Pollen influx was calculated only for the region of the core where ^14^C dates are present.

The opening of COR-4 dates to 815–700 cal. BC, the beginning of the Iron Age in France, and is notable for a number of changes that complicate interpretation of the assemblages. Primary among these is a rising contribution from resistant pollen types such as Lactuceae, Chenopodiaceae and Asteroideae (Fig. 7) and higher indeterminable pollen percentages (Fig. 6) that point to a degree of post depositional biasing [47]. Increased pollen concentrations (Fig. 7) and rising pollen influx (Fig. 9) imply that an element of this may relate to secondary pollen in-wash [15, 54], however, poor preservation conditions are also likely to be responsible. COR-4 records the highest values for *Zygnema*-type (up to 15%), which is associated with shallow seasonally dry lakes, pools and wet areas [55]; conditions that are far from favourable for the preservation of pollen [30]. Ascertaining the extent to which either process is responsible, or if these patterns are solely a result of post-depositional biasing, is problematic. A proportion of the mentioned resistant taxa – such as Lactuceae – probably derive from plants linked with ruderal environments associated with human activity, for which there is strong evidence. Charcoal concentrations reach their highest values in COR-4, AP, likely deriving from trees local to the plateau, remains low, and other apophytes are well represented. Furthermore, increases in AP from trees such as *Fagus*, *Tilia* and in particular *Abies* suggest that the rising pollen influx (Fig. 9) may be a function of a highly open landscape and that in COR-4 the lake is sampling a wider more regional pollen rain [56].

## Discussion

**Fig 10:**
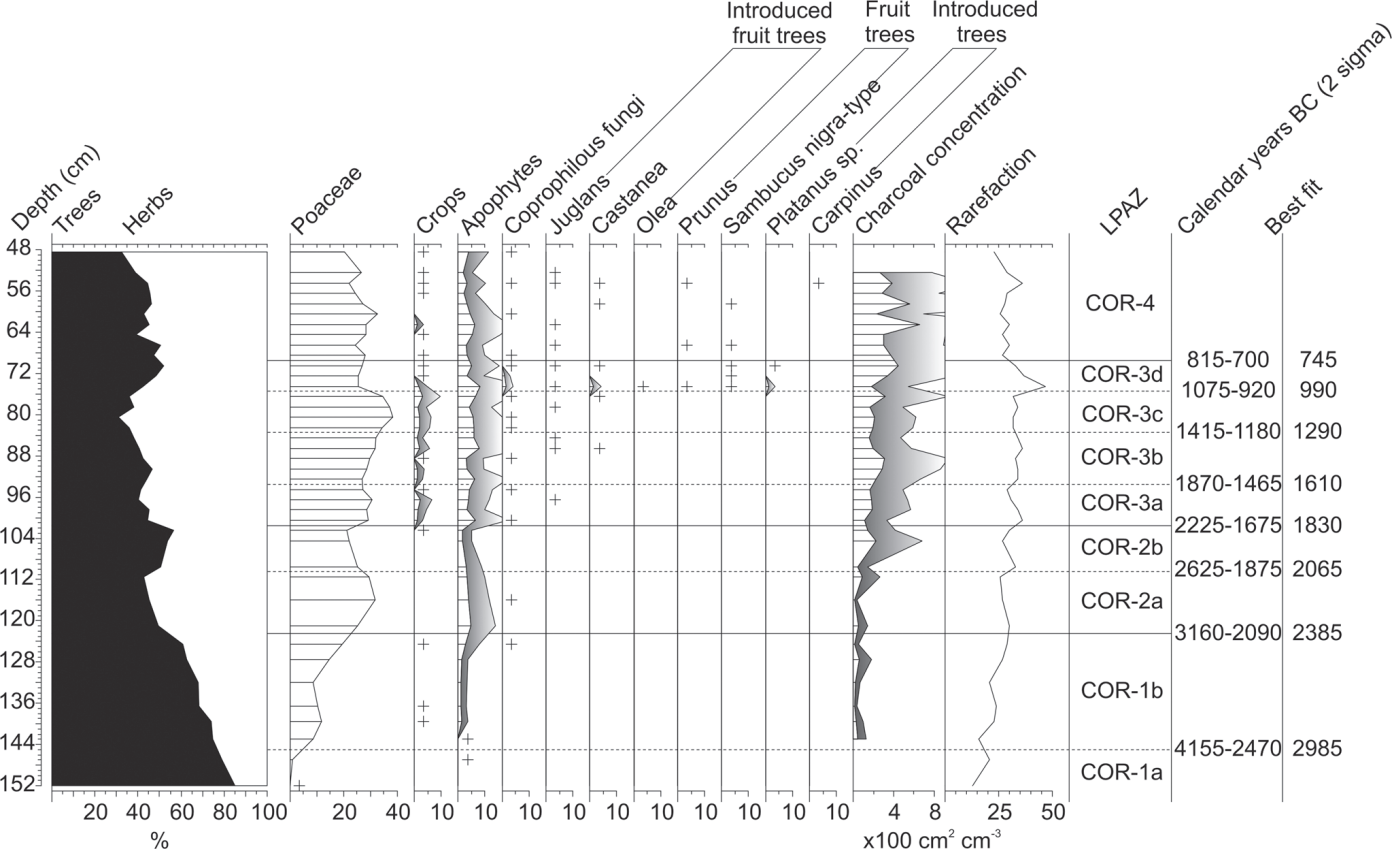
Summary diagram displaying selected taxa, summary curves of crops, apophytes (sum of taxa labeled apophytes in Fig. 7) and coprophilous fungi, Charcoal concentration and rarefaction index are also displayed. + indicates < 1% TLP and where present exaggeration curves are x3. Ages for the LPAZ boundaries reflect two sigma modeled age ranges with the best fit age derived from the smoothed spline CLAM model.

Despite the taphonomic issues associated with the basal section of the cored sequence (Fig. 6), there is a clear signal for two distinct phases of human activity in the Neolithic (Fig. 10). The first is relatively minor, beginning at the opening of COR-1b (4155–2470 cal. BC), and is notable for a slight opening of the landscape and the appearance of apophytes and cereals. The second (COR-2a; opening 3160–2090 cal. BC) reflects an intensification of woodland clearance, although with no indications of agriculture on the plateau. The dates of these events coincide with the archaeological remains of a fortified camp c. 4200–3700 BC and a collective tomb c. 3000–2400 BC [18, 28]. Unfortunately, the imprecise nature of the chronology in this portion of the sequence makes it difficult to determine if the palynological data reflects both or two phases of the same event. Indeed, it is uncertain if the evidence for woodland recovery and declining human activity from the opening of COR-2b (2625–1875 cal. BC) imply a posited period of abandonment, or the aforementioned usage as a cemetery. The age-depth model (Fig. 4, Table 4) allows for either possibility, but increased microscopic charcoal concentrations in COR-2b (Fig. 10), indicative of localised burning, perhaps suggest continued human activity.

From COR-3a (c. 1800 cal. BC) onwards the chronology of the sediment core becomes more certain (Fig. 4, Table 4), and human activity intensifies (Fig. 10) as apophyte contributions rise and crops reach 1–2%. Considering the low pollen productivity and poor dispersal of cereal pollen [30] it is likely these values are indicative of localised agriculture. A slight decline in pollen from crops and apophytes, and minor recovery in woodland occur around 1550 cal. BC, but otherwise agriculture and occupation appears to have been more or less continuous through the Middle Bronze Age. This differs from the archaeological evidence which is scarce during this period and indicative of only small settlements and limited activity on the plateau. Moreover, a posited lull in activity at the beginning of the late Bronze Age (1300–1125 BC, beginning of COR-3c) – inferred from the paucity of material culture dating to this period [28] – is not evident in the palynological data. Instead, agriculture appears to have expanded. COR-3c, the opening of which dates to c. 1290 cal. BC, presents the strongest indications for agriculture in the entire sequence, as woodland pollen falls to its lowest values, indicating an opening of the landscape. By the middle of COR-3c the palynological and archaeological data are more coherent. Pollen from crops reach their maximum values, c. 1030 cal. BC (Fig. 10), when there is archaeological evidence for the beginning of settlement between 1125–950 BC. Yet, this settlement likely reached its peak between 950–800 BC [18, 28], a period represented by COR-3d (beginning c. 950 cal. BC) which is characterised by increased AP and a declining contribution from crops.

In classical palaeoenvironmental studies of Bronze Age human impact from Auvergne, [16] and more widely in central and western Europe [4, 14, 57], such patterns have been interpreted as reflecting periods of abandonment or decline, however this is unlikely to be the case in this instance. Microscopic charcoal values increase and apophytes remain well represented, suggesting a continuing human presence. Moreover, the rarefaction index reaches a peak of 47, indicating a diversification of the flora [41]. In palynological analyses of the development of medieval and industrial era urban environments, apophytes have been shown to be well represented [58, 59] and increased species diversity is associated with the initial phase of development when intense disturbance generates a diverse array of habitats for species to invade [59]. COR-3d may therefore reflect an increasingly dense settlement, or perhaps a nascent urban environment. Indeed, the material evidence suggests the development of an unusually large and dense settlement c. 950–800 BC, with some proto-urban features [28]. The consequent decline in the rarefaction index through COR-3d is also significant as it resembles observations from urban areas. After peaking, diversity has been shown to fall as the initial phase of disturbance is followed by a reduction in the number of available habitats [59].

Aside from strong evidence for agricultural activity a prominent feature of the pollen data from the Bronze Age are the first appearances of a series of non-native taxa (Fig. 10, Table 5). The earliest of these are rare occurrences of *Juglans* and *Castanea* that date to c. 1690 BC (COR-3a) and 1390 BC (COR-3b) respectively (Fig. 7, 10). Typically these trees are associated with the process of Romanisation in France and their pollen is seldom identified prior to this period [14, 16, 19, 60]. In the instances when rare grains are noted, they are commonly attributed to long distance transport from the Mediterranean [11, 61]. This interpretation would seem to hold for these isolated occurrences in the early and middle Bronze Age (COR-3a-b). However, the coeval presence of *Castanea* at 1%, consistent traces of *Juglans*, *Platanus* percentages of 1% and the first appearance of *Olea* (Table 5) at the beginning of COR-3d (c. 950 BC) likely reflect a different origin. Cumulatively these non-native taxa represent over 3% of the assemblage, far in excess of the occasional grains more commonly associated with long distance transport. To consider these observations of *Platanus*, *Castanea* and *Juglans* as long distance transport would require contemporary occurrences of similar values elsewhere in diagrams from Auvergne, but this is not the case (e.g. [16, 19, 20, 21, 22, 23]). Although using pollen evidence alone to evaluate the presence or absence of a species can be troublesome [62], it is reasonable to suggest that percentages of >1% from such as small depositional basin could reflect the local presence of *Castanea* and *Platanus* during the late Bronze Age. The material culture from this period indicates trading links with a number of regions, including those at the periphery of the Mediterranean world [63], and suggests a plausible mechanism for the introduction and spread of these species. The question of whether the continuously rare occurrences of *Juglans* reflect local presence is more complex. Although *Juglans* is frequently cited as a marker of Romanisation in France [19] this interpretation rests on low resolution pollen data from large lakes and mires in mountainous locations [16]; contexts that are far from ideal for detecting small scale localised events [64, 65]. Furthermore, a recent re-appraisal of the palynological evidence from the Jura Mountains and Rhône valley suggests the possibility of an earlier appearance c. 800 BC [66]. Pollen analyses of archaeological sediments from the Limagne plain have also identified *Juglans* from the late Bronze Age [67], thus it is possible these early occurrences at Corent reflect the small scale cultivation of *Juglans* in the Late Bronze Age.

**Table 5:** The depths and dates for the first appearances of pollen from selected plant introductions in the palynological record from Corent. The median probable age from the age-depth model is presented in parentheses.

**Introduction**	**First occurrence**		**Greater than 1%**	
	**Depth (cm)**	**Date (cal. BC)**	**Depth (cm)**	**Date (cal. BC)**
*Juglans*	96.5	2000–1550 (1690)	NA	NA
*Castanea*	86.5	1550–1270 (1390)	74.5	1030–885 (950)
*Olea*	74.5	1030–885 (950)	NA	NA
*Platanus*	74.5	1030–885 (950)	74.5	1030–885 (950)

Across Auvergne the beginning of the Iron Age is characterised by the abandonment of settlement at altitude and a shift towards settlement in the Limagne plain [68, 69]. This pattern is evident in both the archaeological and palaeoecological data from Corent, yet there are also subtle indications of wider changes in human-landscape interactions. COR-4 is notable for a significant increase in pollen from *Abies*, a tree which is tends to be isolated to mountains regions above 1000 m in Auvergne [51]. Its increased representation may reflect some isolated local stands, but more likely it suggests a rising input of regional pollen into the basin, associated with further opening and fragmentation of woodland in the wider landscape [70]. This interpretation is consistent with other studies from Auvergne, which also show increasing deforestation through the Iron Age [16, 21]. However, it is unlikely that the landscape was as open as is frequently implied on the basis of palynological analyses of archaeological contexts [23]. The archaeological samples analysed from Corent – which also date from the Iron Age – record AP percentages between 10–22% (Fig. 6), values similar to sites across Auvergne (e.g. [23, 67]), but far below the 32–50% recorded in the core. These results clearly indicate that the case made for a widely deforested landscape in the Iron Age is overstated. *Quercus* percentages and influx are also higher in COR-4 which may relate to the aforementioned process. However, archaeobotanical data from the Limagne plain show a shift away from Oak exploitation through the Iron Age [71] and it may be the case that the palynological data reflect preferential exploitation of other tree species such as *Fagus*.

Despite clear archaeological evidence for two periods of settlement on the plateau during the Iron Age these are difficult to trace through the palaeoecological record. The first is noted between c. 600–550 BC, but at lower density than in the Late Bronze Age [28]. This phase (occurring c. 62 cm) is poorly resolved in the palynological data with only a marginal increase in apophyte and crop pollen c. 450 BC, suggestive of agricultural activity. The archaeological remains suggest that this settlement was destroyed by fire [28] and it is worthwhile to note that microscopic charcoal concentrations attain peak values for the entire core at this time. The second phase of Iron Age activity is related to the emergence of the Oppidum of Corent at c. 125 BC [18], around 54 cm in the sediment core. The pollen influx of introduced taxa (such as *Castanea* and *Juglans*) peak at levels comparable to those noted in the Late Bronze Age (Fig. 9) and again may imply the presence of these fruit trees. Material culture associated with the Mediterranean world is exceedingly well represented in this period [18] and it possible that *Juglans* and *Castanea* records reflect palynological evidence for this trade and contact. Aside from fruit trees, the pollen spectrum from this depth is characterised by a minor increase in apophytes and traces of pollen from crops (Fig. 10). These data suggest that the urban character of the Oppidum was not agricultural, nor do they imply a particularly large urban settlement. A peak in the rarefaction index again suggests increased floristic diversity, as has been noted in other urban areas [59], but the intensity is significantly lower than that noted from COR-3d (Fig. 10). Given the scale of the Oppidum, which comprised housing, administrative, commercial and religious buildings [18], these data are somewhat unusual. One possible explanation may be that there are biases in the pollen assemblages from this time period. Both resistant taxa (c.40–50%) and Indeterminable pollen are particularly high (c. 30–45%) in the latter part of COR-4 (Fig. 6), which suggests post-depositional biasing or perhaps increased deposition of secondary pollen. Considering the concurrent increases in total pollen influx and *Glomus* percentages (Fig 7, 10), it seems plausible that a portion of this increased influx relates the erosion and re-deposition of ‘old’ secondary pollen from soils in the catchment [15]. This explanation is the more likely given that intense urban settlement is associated with increased erosion and delivery of pollen to depositional basins [58]. Nevertheless, it is important to note that the large increases in the resistant taxa grouping are a result of a rising contribution from Lactuceae, Asteroideae and Chenopodiaceae. Although these pollen types are highly resistant to corrosion and can have long residence times in soils [48], they are also produced by ruderal plants of the dandelion and goosefoot families. Therefore, it is likely that the apophyte curve somewhat underestimates the contribution of cultural plants – and hence the scale of human impact – for this period of the core.

## Conclusions

Archaeological thought concerning urbanism in Central and Western Europe is currently in a state of flux, with recent research challenging previous ideas of how and when urban areas developed [10, 72, 73]. Characterising and tracing the development of urban areas is a far from simple task and there is much debate surrounding what constitutes an urban setting and the complex irregular evolution of such areas [74, 75]. Through high resolution palaeoecological analyses of a small depositional basin – located within the archaeological remains of the Oppidum of Corent – this paper has explored how environmental archaeology can contribute to the understanding of the development of urban environments. In adopting a long term perspective it has been possible to identify the rhythms and intensity of various phases of human activity from the Neolithic to the Roman conquest. In turn, this has allowed comparisons to be drawn between the various periods of settlement on the Corent Plateau. Early activity (Neolithic to Middle Bronze Age) was primarily characterised by an opening of the landscape and a gradual increase in the intensity of agriculture, while data from the Late Bronze Age and La Tène show significant differences. The key features of these latter periods are increased biodiversity, plants indicative of ruderal environments and limited evidence of agriculture, which resemble patterns observed in palaeoecological studies of other urban areas. Both periods are also notable for the early occurrences of non-native plant taxa, which could be considered a palynological fingerprint of long distance trade, and contact, especially with the Mediterranean world.

Small depositional basins record highly localised information and when intimately associated with archaeological remains they can reveal information regarding human land use that is lacking from conventional palaeoecological studies. By exploiting this potential, this study has provided observations that contribute to the debate surrounding the non-linear, erratic nature of proto-urbanisation in Western and Central Europe. The observation of different palynological signatures for the Late Bronze Age, La Tene, and earlier periods illustrate how environmental archaeology can furnish insights complementary to the material remains more commonly the focus of archaeological attention. Indeed, this paper has also provided a long term perspective allowing the identification of the patterns and rhythms of human activity that are poorly represented in the archaeological record.

## Supporting Information

S1 Dataset Pollen, microscopic and radiocarbon data that form the basis of this manuscript. (XLSX)
